# Formulation of lipid polymer hybrid nanoparticles of the phytochemical Fisetin and its in vivo assessment against severe acute pancreatitis

**DOI:** 10.1038/s41598-023-46215-8

**Published:** 2023-11-04

**Authors:** Randa Hanie Awadeen, Mariza Fouad Boughdady, Randa A. Zaghloul, Wael M. Elsaed, Irhan Ibrahim Abu Hashim, Mahasen Mohamed Meshali

**Affiliations:** 1 Department of Pharmaceutics, Faculty of Pharmacy, Mansoura University, El-Gomhoria Street, Mansoura, 35516 Dakahlia Egypt; 2 Department of Biochemistry, Faculty of Pharmacy, Mansoura University, Mansoura, 35516 Egypt; 3 Department of Anatomy and Embryology, Faculty of Medicine, Mansoura University, Mansoura, 35516 Egypt

**Keywords:** Biochemistry, Nanoscience and technology

## Abstract

Fisetin (FST) is a naturally occurring flavonol that has recently emerged as a bioactive phytochemical with an impressive array of biological activities. To the author knowledge, boosting the activity of FST against severe acute pancreatitis (SAP) through a nanostructured delivery system (Nanophytomedicine) has not been achieved before. Thereupon, FST-loaded lipid polymer hybrid nanoparticles (FST-loaded LPHNPs) were prepared through conjoined ultrasonication and double emulsion (w/o/w) techniques. Comprehensive in vitro and in vivo evaluations were conducted. The optimized nanoparticle formula displayed a high entrapment efficiency % of 61.76 ± 1.254%, high loading capacity % of 32.18 ± 0.734, low particle size of 125.39 ± 0.924 nm, low particle size distribution of 0.357 ± 0.012, high zeta potential of + 30.16 ± 1.416 mV, and high mucoadhesive strength of 35.64 ± 0.548%. In addition, it exhibited a sustained in vitro release pattern of FST. In the in vivo study, oral pre-treatment of FST-loaded LPHNPs protected against l-arginine induced SAP and multiple organ injuries in rats compared to both FST alone and plain LPHNPs, as well as the untreated group, proven by both biochemical studies, that included both amylase and lipase activities, and histochemical studies of pancreas, liver, kidney and lungs. Therefore, the study could conclude the potential efficacy of the novel phytopharmaceutical delivery system of FST as a prophylactic regimen for SAP and consequently, associated multiple organ injuries.

## Introduction

Phytomedicines have been considered a mainstream health approach to avoid the side effects caused by synthetic drugs. However, the main downfall of this approach is the low bioavailability associated with those phytomedicines, due to the fact of their poor aqueous solubility, possible first-pass metabolism, and instability by the conventional drug formulation^1^. As a result, Nanophytomedicines have been sought by combining biologically active phytochemicals with nanotechnology through numerous cutting-edge delivery systems.

Of these systems, polymeric nanoparticles and lipid-based ones have been considered the most efficient drug carriers for bioavailability enhancement. For instant, they allow a sustained release of drugs in the gastrointestinal tract (GIT), improved nanoscopic dimensions, and bioadhesion^2^. In addition, lipid based delivery systems have excellent biocompatibility and biomimetic nature, so, they exhibit multi-fold applications in tumor targeting, gene delivery, and macrophage targeting^3^. Unfortunately, lipid-based delivery systems alone suffer from reduced biological as well as physico-chemical stability, high polydispersity, biphasic release, narrow scope of surface functionalization, insufficient content integrity, and drug expulsion during storage^4^. Conversely, polymeric nanoparticles reveal better stability and structural integrity, minimized drug leakage and burst release, sustained plasma circulation, reduced polydispersity, imparting stimuli responsiveness and site specificity. But like lipid-based delivery systems, they exhibit certain disadvantages such as, the drug loading and entrapment efficiency for hydrophilic drugs are low, organic solvents exposure, toxicity and biocompatibility problems^5^.

In a nutshell, both systems (lipid and polymer-based ones) possess distinctive features, but suffer from certain demerits individually. Integration of both systems could potentially synergize their unique features and overcome the difficulties related to each system alone. This hybrid endeavor is termed lipid-polymer hybrid nanoparticles (LPHNPs) or lipomer^6^. LPHNPs can be sub-categorized regarding their structure into: self-emulsifying, lipid-shell polymer core, polymer-shell lipid-core, and matrix-structured LPH. The processing technique and the polymer lipid composition in the formulation are considered the building block that control the nanostructure of the systems.

Natural polymers offer advantages such as biocompatibility, biodegradability, low cost and low toxicity, thus making them top-notch candidates to be enclosed in the lipid systems. Amongst them, chitosan (CT) is the most frequently used biopolymer within lipid systems. CT-based LPHNPs result in polymer-shell lipid-core structures with complete CT surface coatings. They are superior to other biopolymer-lipid systems regarding mucoadhesion and absorption-enhancing properties. Consequently, cellular uptake can be improved in comparison with the precursor uncoated lipid systems, resulting in higher drug transport across the intestinal epithelium^7^.

**Figure 1:**
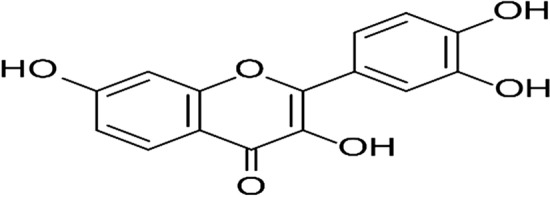
Structural formula of FST.

Fisetin (3,7,3′,4′-tetrahydroxyflavone, FST) (Fig. 1) is a naturally occurring flavonol with potential anti-inflammatory, antioxidant, antiangiogenic, hypoglycemic, hypolipidemic, neuroprotective, senotherapeutic and antitumor effects^8^. Unfortunately, FST is a class II drug with low aqueous solubility and high permeability^9^. Therefore, it is imperative to develop suitable drug delivery systems to enhance the oral delivery of FST as well as its therapeutic efficacy^10^.

Pancreatic cancer is considered one of the top five fatal malignancies. Therefore, it is important to prevent and early diagnose this type of cancer. Following acute pancreatitis (AP), a ninefold increased risk of pancreatic cancer has been reported. Recurrent episodes of severe acute pancreatitis (SAP) has been suggested to cause chronic pancreatitis which could progress to pancreatic cancer^11^.

To the author knowledge, boosting the activity of FST against severe acute pancreatitis through LPHNPs has not been achieved before. Therefore, the current study was mapped out to prepare an oral FST-loaded LPHNPs (based on the formation of w/o/w double emulsion using Gelucire as the lipid phase and coated with CT in the external aqueous phase) to achieve two main objectives. The first one was to improve the poor aqueous solubility of FST by dissolving in a Tromethamine (Tris) solution. The second objective aimed to explore the possible prophylactic anti-inflammatory effect of FST-loaded LPHNPs against l-arginine-induced severe acute pancreatitis (SAP) in rats. The net result would be the development of FST-loaded LPHNPs delivery system that could offer enhanced dissolution and pharmacological effect.

## Material and methods

### Materials

FST and low molecular weight (M.Wt) CT [(M.Wt of 50–90 KDa), and degree of deacetylation 93%], and Tromethamine (Tris (hydroxymethyl) aminomethane) were obtained from (Sigma-Aldrich, chemical Co., St. Louis, Mo., USA). Labrasol and Gelucire (50/13, pellets) were kindly supplied by (Gattefossé-SAINT-PRIEST, Cedex, France). Tween 80 was purchased from (El Gomhoria Co., Cairo, Egypt). Potassium dihydrogen phosphate, disodium hydrogen phosphate, methanol, and Acetic acid were purchased from (EL-Nasr pharmaceutical chemicals Co., Cairo, Egypt). l-arginine was purchased from (AL-Molok chemicals Co., Cairo, Egypt). Commercially available kits for in vivo study were purchased from BioMerieux, France, and Bio-diagnostic Co., Cairo, Egypt.

### Solubility study of FST

Solubility study of FST was carried out in deionized water (DW) and Tris solutions of different concentrations. Excess FST amount (50 mg) was dispersed in 5 mL of either DW or Tris solution and placed in screw-capped glass vials. These vials were put in a thermostatically controlled water bath at (37 ± 1 °C) (Grant Instrument, Cambridge Ltd., UK) and shaken continuously for 48 h. Subsequently, the vials were removed and the solutions were filtered using a 0.45 µm membrane filter. The amount of the dissolved FST was spectrophotometrically measured at 358 nm^12^ using an Ultraviolet–Visible (UV–VIS) spectrophotometer (Shimadzu; UV-150-02, Sersakusho; Ltd, Kyoto, Japan).

### Preparation of FST-loaded LPHNPs

FST-loaded LPHNPs were prepared by a combination of two techniques (ultrasonication and w/o/w double emulsion). The composition of different LPHNPs formulae is shown in Table 1. Accordingly, a clear uniform oil phase (o) was formed by heating an accurately weighed amount of Gelucire 50/13 (1.5 g) and Labrasol (0.2 mL) at 10–15 °C above Gelucire melting point (50 °C). A constant drug amount (2 mg) was dissolved in 1 mL of Tris solution (6% w/v) via sonication in a water bath for 15 min to form the internal aqueous phase (w_1_). Afterward, the internal aqueous phase (w_1_) was added to the formerly melted oil (o) at the same temperature. Solidification with size reduction of the prepared mixture was rapidly initiated in ice bath via ultrasonic homogenizer (3 min at 90% amplitude, Pulser; 1 s ON/1 s OFF) to acquire a primary (w_1_/o) emulsion. Subsequently, 10 mL of CT solution (external aqueous phase, w_2_) with different concentrations (0.1, 0.2, and 0.3% w/v), dissolved in 2% acetic acid, were added to the primary w_1_/o emulsion employing ultrasonic homogenization in ice bath (10 min at 90% amplitude, Pulser: 1 s ON/1 s OFF) to form w_1_/o/w_2_ double emulsion. An uncoated formula containing water instead of CT solution in the external aqueous phase was prepared similarly for comparison. The acquired formula was magnetically stirred for 30 min at room temperature using a magnetic stirrer (Magnetic stirrer; MS300HS, MTOPS Corp., Korea). Then, the FST-loaded LPHNPs were collected via cooling centrifugation (90 min at 4 °C and 13,000 rpm) (Cooling Centrifuge; CE16-4X100RD, ACCULAB, USA) and exposed to freeze drying under vacuum at − 80 °C (Freeze dryer; SIM FD8-8T, SIM international, USA) to gain the final lyophilized product that was kept at 4 °C for forthcoming assessment.

**Table 1:** Composition of FST-loaded LPHNPs formulations.

Formula code	Formulae composition			
	FST (mg)	Gelucire (g)	Labrasol (mL)	CT conc. (%w/v)
F1 (Uncoated formula)	2	1.5	0.2	–
F2	2	1.5	0.2	0.1
F3	2	1.5	0.2	0.2
F4	2	1.5	0.2	0.3

### Characterization and optimization of FST-loaded LPHNPs formulations

#### Entrapment efficiency percent (EE %) and loading capacity percent (LC %)

The entrapment efficiency % of FST-loaded LPHNPs was indirectly estimated by determining the unentrapped drug amount in the supernatant after cooling centrifugation (13,000 rpm at 4 °C for 90 min)^13^. The supernatant was suitably diluted and the absorbance was spectrophotometrically measured at 358 nm using UV–VIS spectrophotometer. The blank was the supernatant of the plain LPHNPs. The EE % was calculated from Eq. (1). The isolated LPHNPs was freeze dried and weighed, then, the loading capacity percent (LC %) was calculated using Eq. (2)^14^.

1 $${\text{EE}}\,\% = \frac{{{\text{FST total amount}} - {\text{FST un - entrapped amount}}}} {{{\text{FST total amount}}}} \times 100$$

2 $$\text{LC}\,\%=\frac{\text{Entrapped FST amount }}{\text{Weight of the dried NPs}} \times 100$$

#### Particle size (PS) and particle size distribution (PDI)

Particle size (PS) as well as polydispersity index (PDI) are significant characteristics that affect the distribution of NPs. For the freshly prepared NP formulations, the average PS and PDI were measured using Zetasizer Nano ZS (Malvern Instruments; Malvern, UK). The analysis was carried out after proper dilution with DW^15^.

#### Zeta potential (ZP)

ZP is a crucial feature estimating the nano-particulate system stability. It determines the charge on the surface of particles by perceiving the electrophoretic mobility in the electrical field. Using the Zetasizer Nano ZS (Malvern Instruments, Malvern, UK), ZP measurement was performed on the freshly prepared NPs after suitable dilution with DW^16^.

#### Mucoadhesive strength

The mucin-binding efficiency (%) is an indicator For the mucoadhesive strength of the LPHNPs. Concerning mucoadhesive strength, all FST-loaded LPHNPs were estimated depending on the interaction between CT-coated LPHNPs with a positive charge and mucin with a negative one. Concisely, equal volumes (5 mL) of mucin (0.5 mg/mL in phosphate-buffered saline of pH 7.4) as well as dispersion of NPs From each formula were vortexed (vortex mixer mod. VM-300P, Teiwan associated with Cannic. Inc., USA). Subsequently, the dispersion was shaken for 1h at 100 rpm and 37°C using a thermostatically controlled shaking incubator (GFL Gesellschaft für Labortechnik; Burgwedel, Germany) and centrifuged for 1 h at 10,000 rpm (Sigma, D-37520, Germany). The amount of free mucin in the obtained supernatant was measured spectrophotometrically at 258 nm using a UV–VIS spectrophotometer. The mucin-binding efficiency (%) was determined using Eq. (3)^17^:

3 $$\text{Mucin-binding efficiency}(\%) =\frac{Total\, amount\, of\,mucin-Free\, amount\, of\, mucin}{Total\, amount\, of\, mucin}\times 100$$

### Evaluation of the optimized FST-loaded LPHNPs formula (F3)

#### Transmission electron microscope (TEM)

Morphological examinations of the optimized LPHNPs (F3) and the uncoated NPs (F1) were carried out through TEM. Briefly, one mL of the NP suspension was diluted ten times with DW. A drop of the diluted suspension was put on the surface of carbon-coated copper grids (200 mesh; Science Services; Munich, Germany). The extra material was subsequently detached using a filter paper to leave a thin film that was extended over the holes and left for 10 min at room temperature to dry completely before examination^18^. Capture as well as image analysis were done by digital micrograph and soft imaging viewer software.

#### Fourier transform-infrared spectroscopy (FT-IR)

The FT-IR spectra of FST, Tris, CT, Gelucire, physical mixtures regarding the optimized coated LPHNPs (F3) and the optimized freeze-dried coated LPHNPs (F3) as well as its equivalent plain coated formula were detected via FT-IR Spectrophotometer (Madison instruments; Middleton, WI, USA). A hydrostatic press was adopted to prepare potassium bromide discs. The scanning range was from 500 to 4000 cm^−1^^18^.

#### Differential scanning calorimetry (DSC)

DSC was carried out through a Differential scanning calorimeter (DSC-60, Shimadzu Corporation, Japan) (standardized with indium (m.p = 156.6 °C, purity of 99.99%), heating rate = 10 °C/min). FST (4 mg), Tris, CT, Gelucire, physical mixtures regarding the optimized coated LPHNPs (F3) and the optimized freeze-dried coated LPHNPs (F3) and its corresponding plain coated one were heated (in the range of 30—450 °C) in aluminum crimped pans under nitrogen gas flow (20 mL/min). An empty pan was similarly sealed to be used as a reference^15^.

#### X-ray diffractometry (XRD)

X-ray diffraction patterns of FST, Tris, CT, Gelucire, physical mixtures regarding the optimized coated LPHNPs (F3) and the optimized freeze-dried coated LPHNPs (F3) and its corresponding plain coated one were recorded via X-ray diffractometer equipped with Cu-Kα (Diano Corp., Woburn, MA, USA). The analysis was done at a 2θ angle (scanning range from 3° to 50°) as well as at a voltage and a current of 45 kV and of 9 mA, respectively^17^.

### In vitro release study

The in vitro release studies of FST from the prepared CT-coated LPHNPs (F3) and the uncoated formula (F1) compared to its diffusion from FST aqueous dispersion and FST/Tris solution containing 6%w/v Tris (S1 and S2, respectively) were examined. The study was carried out in different release media simulating gastrointestinal fluids (HCl (pH 1.2) and phosphate buffer (pH 6.8 and 7.4)) via vertical Franz diffusion cells having a surface area of 7.07 cm^2^. A spectrapor® membrane (MW cutoff 12,000–14,000 Da; Spectrum Medical Industries Inc., LA, USA) was equated with release medium overnight and used to separate the donor compartment and the receptor one. Briefly, the NPs (either F3 or F1; containing an equivalent FST amount (2 mg)) were dispersed in DW. Each dispersion was placed in the donor compartment, while a 50 mL release medium was placed in the receptor one. Likewise, the FST dispersion in DW or FST/Tris solution (containing 6%w/v Tris) with an equivalent FST amount (2 mg) was treated similarly. The whole diffusion cells assembly were shaken (100 rpm/min, 37 ± 0.5 °C) using a thermostatically controlled shaking incubator (GFL Gesellschaft für Labortechnik; Burgwedel, Germany). At different time intervals, 3 mL samples were taken from the receptor compartment and replaced with equal volume of fresh medium that was maintained at 37 ± 0.5 °C. Filtration of samples was done using a 0.45 µm membrane filter (EMD Millipore; Billerica, MA, USA) and the amount of the released FST was spectrophotometrically assessed at 358 nm. Finally, the cumulative percent released of FST was calculated and plotted against time^17^.

### Kinetic analysis

To acquire a deep perception of the mechanism of drug release from NPs, the obtained in vitro release results of the chosen formulations (F3 and F1), FST dispersion in DW (S1), and FST/Tris solution (S2) were fitted to diverse kinetic models encompassing zero and first-order^19^, Higuchi diffusion equation^20^. Besides, the Korsmeyer-Peppas kinetic model was applied for the first 60% release data to confirm the mechanism of release^21^. Moreover, Weibull model (an empirical model) that represents the different release patterns from LPHNPs, was applied to the release data^22^. The model offering the highest coefficient of determination (R^2^) is regarded the predominant model^23^.

### Stability study of the optimized FST-loaded LPHNPs (F3)

Adopting the International Council for Harmonisation (ICH) guidelines, stability study for the freshly prepared FST-loaded LPHNPs aqueous dispersions of the optimized formula (F3) was performed. LPHNPs aqueous dispersions were filled into hermetically sealed glass bottles and keept for 6 months at both ambient and refrigerated (4 ± 1 °C) conditions with no agitation or stirring^15^. The stability of the optimal LPHNPs (F3) was assessed concerning physical appearance, PS, ZP, PDI, and drug retention %; initially at zero time (at the preparation day) as well as after storage for 1, 2, 3, 4, 5 and 6 months. Drug retention % was estimated using Eq. (4):

4 $$\text{Drug retention} (\%)=\frac{EE\,\%\,at \,each\,time\,interval}{EE\,\%\,initial}\times 100$$

### In vivo assessment studies of the optimized FST-loaded LPHNPs (F3)

#### In vivo induction of SAP and multiple organ injuries

The study recruited thirty healthy adult male Sprague–Dawley rats (200 ± 5 g, age 7–8 weeks), that were housed in the Animal House, Faculty of Pharmacy, Mansoura University, Mansoura, Egypt. Initially, rats were randomly divided in cages (six rats per cage) to allow acclimation and observe any behavioral or health anomalies. Standard conditions of animal care (steady light/dark cycles, temperature (25 ± 2 °C), and free access to water and food) were maintained throughout the experiment. The study protocol was approved by the ethical committee of the Faculty of Pharmacy, Mansoura University, Mansoura, Egypt, (code number: 2022–224). The study was carried out according to the Principles of Laboratory Animal Care, National Materials Institute of Health Publication (No. 85–23, revised 1985) and ARRIVE guidelines.

The following groups were assigned (n = 6) and orally received the following for seven successive days using an intragastric tube;

Group I: Normal control group; rats received normal saline only.

Group II: l-arginine (l-arg) group; rats received normal saline only.

Group III: Pure FST group; rats received FST (10 mg/Kg/day) suspended in 0.5% sodium carboxy methyl cellulose (CMC)^24^.

Group IV: Plain LPHNPs group; rats received Plain LPHNPs (un-loaded F3) as lyophilized powder suspended in 0.5% sodium CMC.

Group V: The optimal FST-loaded LPHNPs (F3) group; rats received FST-loaded LPHNPs (F3) containing FST equivalent to (10 mg/Kg/day) as lyophilized powder suspended in 0.5% sodium CMC.

On the 7th day, an hour after the last treatment of the above different groups, SAP was induced in groups (II-V) by twice intraperitoneal (i.p.) injections of l-arg (250 mg/100 g body weight) with an interval of one hour^25^.

#### Collection of the biological samples

After twenty-four hours from the last l-arg dose, rats were anesthetized using thiopental sodium (40 mg/kg, i.p). Blood samples were collected from the retro-orbital venous plexus, and left for two hours-stand at room temperature. Subsequently, they were centrifuged for 15 min at 4 °C and 3000 rpm; to collect their sera for the estimation of all biochemical parameters. Sera were directly utilized for quantification of C-reactive protein (CRP) (Tina-quant C-reactive protein Gen.3, Roche Diagnostics, USA). Then, the scarification of rats was done by neck dislocation. For immunohistochemical (IHC) and histopathological examination, the following organs (pancreas, liver, lung, and kidney) were removed and blot-dried; then, a part of each organ was immersed in buffered formalin (10%) and fixed in paraffin wax. The remaining pancreatic tissue was either preserved at − 80 °C for ELISA analysis or homogenized with phosphate-buffered saline (PBS, pH 7.4) and centrifuged at 4 °C (4000 rpm) to collect the supernatant for oxidative stress assessment. The total proteins in the homogenates were estimated using commercially available kits (Bio-diagnostic Co., Cairo, Egypt); following the manufacturer’s directions and kept at − 80 °C for forthcoming analysis^26^.

#### Measurement of serum biomarkers

Sera samples were used for the spectrophotometric assessment of the activity of lipase, amylase, alanine aminotransferase (ALT), aspartate aminotransferase (AST), and creatinine level by commercially available kits (ZL-219 001, 281 001, 263 001, 259 001 and 235 001, respectively, Spectrum Diagnostics, Cairo, Egypt) following the instructions of the manufacturers^27^.

#### Measurement of oxidative stress/anti-oxidants biomarkers

Pancreatic homogenate samples were used for the spectrophotometric assessment of the malondialdehyde (MDA) (a lipid peroxidation end product) and reduced glutathione (GSH) levels by commercially available kits (MD 25 29 and GR 25 11, respectively Bio-diagnostic co., Giza, Egypt) following the instructions of the manufacturer^27^.

#### Measurement of inflammatory biomarkers

Pancreatic homogenate samples were used for the evaluation of rat nucleotide-binding oligomerization domain, leucine-rich repeat and pyrin domain-containing protein 3 (NLRP3) (Cat. No. KCD04232, Beijing AVIVA Systems Biology co., China), TNF-α (Cat. No abx050220, Abbexa, Ca, UK), IL-1β and IL-6 (Cat. No SEA563Ra and SEA079Ra, respectively, Cloud-clone Corp., TX, USA) by commercially available ELISA kits, following the instructions of the manufacturer.

#### Histopathological and immunohistochemical (IHC) examination of Toll-like receptor 4 (TLR4) and nuclear factor kappa B (NF-κB)

Organs that were preserved in paraffin wax, were cut into four μm-thick coded sections and stained with hematoxylin and eosin (H&E) stain. Histological alterations were detected and photographed via an Olympus® digital camera connected to an Olympus® light microscope (Shinjuku Co., Tokyo, Japan) by a skilled pathologist who was blind to the coding system of the different groups. Histopathological scorings were done as previously described by others for the pancreas^28^, liver^29^, lung^30^, and kidney^31^.

Treatment of two diverse sets of deparaffinized pancreatic sections was carried out with an IHC staining Kit (Invitrogen™; Ca, USA), following the protocol of the manufacturer. Consequently, each group was incubated overnight with a different primary antibody; TLR4 (Wuhan Servicebio Biotechnology, Wuhan, China, dilution 1:500) and NF-κB (ABclonal (Woburn, MA, USA) at dilution 1:200), following the protocol of each primary antibody. Then, sections were incubated with horseradish peroxidase-conjugated secondary antibodies and visualized by 3,3′-diaminobenzidine tetrahydrochloride (Genemed; Biotechnologies Inc., USA). Finally, sections were counterstained with Mayer's hematoxylin and examined using ImageJ software version K 1.45. The parameters % area were measured using five randomly-taken non-overlapping fields (× 40)^26^.

### Statistical analysis

The in vitro data were represented as mean ± standard deviation (SD). On the other hand, the in vivo data were displayed as mean ± standard error of the mean (SEM) or median and interquartile change as appropriate. The differences between groups were assessed by one-way analysis of variance (ANOVA) followed by Tukey’s post-hoc test for multiple comparisons. Statistical analysis for non-parametric scoring data was carried out using the Kruskal–Wallis test followed by Dunn multiple comparison test. Statistical evaluation of the results was carried out by Graph Pad Prism version 8.0.2 (La Jolla, CA, USA).

### Ethical approval

The study protocol was approved by the ethical committee of the Faculty of Pharmacy, Mansoura University, Mansoura, Egypt, (code number: 2022-224). The study was carried out according to the Principles of Laboratory Animal Care, National Materials Institute of Health Publication (No. 85-23, revised 1985) and ARRIVE guidelines.

### Informed consent

We submit the manuscript entitled “Formulation of lipid polymer hybrid nanoparticles of the phytochemical Fisetin and its in vivo assessment against severe acute pancreatitis”.  All authors would like to have it considered for publication in Scientific Reports. We hope our manuscript will have the opportunity to be published in it.

## Results and discussion

### Solubility study of FST

A ninefold increment in FST solubility (4.5 mg/mL) was observed upon using 6% w/v Tris solution compared to its solubility in water. It could be speculated that Tris increased the pH in the microenvironment around FST particles due to its comparatively basic nature, hence higher solubility of FST was observed^32^. From the aforementioned result, Tris was chosen as the internal aqueous phase during LPHNPs preparation.

### Characterization and optimization of FST-loaded LPHNPs formulations

#### entrapment efficiency Percent (EE %) and loading capacity percent (LC %)

The EE % is picked out as an indicator of the reproducibility and efficacy of the proceeding technique. The EE % ranged from 46.29 ± 0.552 (uncoated F1) to 64.04 ± 0.622 (coated LPHNPs) (Table 2). It can be deduced from Table 2 that the EE % increased with increasing CT concentration. It might be expected that increasing CT concentration leads to increased viscosity, with a consequent decrease in drug leakage from the LPHNPs matrix; hence, increasing the EE %. Moreover, as the polymer concentration increases, the amount of the formed LPHNPs increases with successive increments in the encapsulated FST amount. These results agreed with the reported studies^33^. A profound look to Table 2 revealed that the data of LC % of the LPHNPs formulae ranged from 24.17 ± 0.357 (uncoated F1) to 33.44 ± 0.827 (coated LPHNPs; F4). These results concurred with those of EE % based on the explanation of the increment of CT concentration in the investigated formulae.

**Table 2:** Characterization of FST-loaded LPHNPs formulations (See Table 1 for composition). Each value represents the mean ± S.D (n = 3).

Formula code	EE %	LC %	PS (nm)	PDI	ZP (mV)	Mucin binding efficiency %
F1	46.29 ± 0.552	24.17 ± 0.357	78.53 ± 0.573	0.429 ± 0.005	− 17.13 ± 0.634	–
F2	51.35 ± 1.607	26.81 ± 1.238	86.36 ± 0.858	0.334 ± 0.028	+ 23.13 ± 1.054	31.52 ± 0.914
F3	61.76 ± 1.254	32.18 ± 0.734	125.39 ± 0.924	0.357 ± 0.012	+ 30.16 ± 1.416	35.64 ± 0.548
F4	64.04 ± 0.622	33.44 ± 0.827	407.57 ± 0.939	0.418 ± 0.008	+ 33.53 ± 1.321	39.08 ± 0.567

#### Particle size (PS) analysis and polydispersity index (PDI)

Regarding LPHNPs size, both the average PS (nm) and their size variability (PDI) are crucial factors for evaluating the colloidal dosage form stability during storage under different conditions^34^. PS influences the therapeutic efficacy as it is one of the leading factors in determining the uptake of NPs by the epithelial and mucosal tissue. Besides, PDI (a dimensionless index) which expresses the relative divergence of the particle size distribution affects the therapeutic performance of the NPs formulations.

Careful examination of Table 2 revealed that all formulae exhibit small PDI values (< 0.5) which points out a homogenous particle size distribution. Uniform particle size distribution helps in maintaining the colloidal dosage form stability without precipitation or formation of microparticles^5^. Furthermore, Table 2 shows that CT concentration has a conspicuous effect on PS; a substantial increment in PS (from 86.36 ± 0.858 to 407.57 ± 0.939) is clear with increasing the CT concentration. This might be ascribed to increasing the aqueous phase viscosity with subsequent increase in the PS^35^.

#### Zeta potential (ZP)

ZP is a prime sign for NPs that strongly affects their physical stability, cellular uptake, and mucoadhesive ability. It points out the repulsion degree between close as well as correspondingly charged particles in the dispersion which reflects the prevention of particle aggregation. As a rule of thumb, a good stability of NPs occurs when ZP value is above + 30 mV or below − 30 mV^15,17^.

Herein from Table 2, it is noted that ZP value of the uncoated formula (F1) is − 17.13 ± 0.634. However, it turns over to positive values for the coated LPHNPs and ranges from 23.13 ± 1.054 to 33.53 ± 1.321. The prospective explication is that surface modification of the NPs by coating with CT provideed freely ionized amino groups (NH3^+^) on their surface. This led to more electrostatic repulsion between NPs with successive improvement in the nanodispersion stability^36^.

#### Mucoadhesive strength

Mucoadhesive characteristics provide a significant impact on augmenting the scope of LPHNPs application. In this work, FST-loaded LPHNPs revealed a distinct increment in the mucin-binding efficiency (%) (From 31.52 ± 0.914 to 39.08 ± 0.567%) accompanying the increase in the ZP values as demonstrated in Table 2. CT with its mucoadhesive properties would help the electrostatic interaction between positively charged amino groups of CT and negatively charged sialic acid groups of mucin^36^, thus fostering the cellular uptake of NPs; leading to efficient delivery of therapeutics^17^.

**Figure 2:**
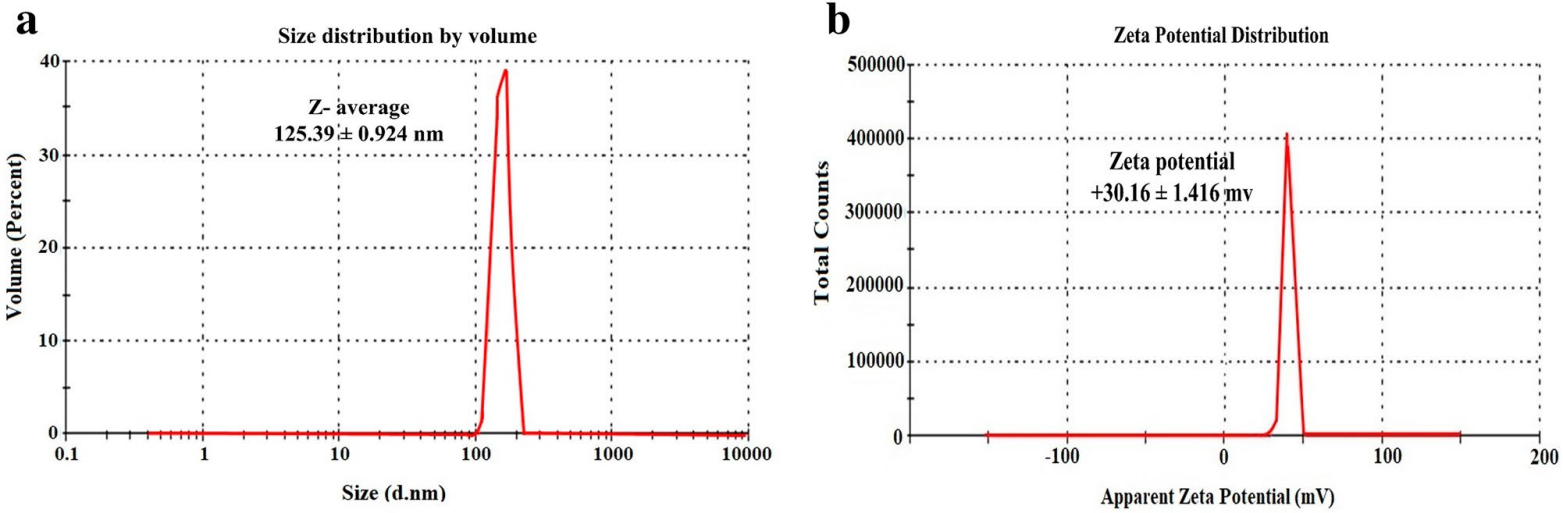
Particle size (**a**) and zeta potential (**b**) of the optimized FST-loaded LPHNPs (F3).

In conclusion, FST-loaded LPHNPs formula (F3) composed of (Gelucire (1.5 g), Labrasol (0.2 mL), and CT (0.2% w/v)) was chosen for the forthcoming evaluations. This was based on its high EE (%) (61.76 ± 1.254%), proper LC % (32.18 ± 0.734%), low PS (125.39 ± 0.924 nm) (Fig. 2a), low PDI (0.357 ± 0.012), high ZP (+ 30.16 ± 1.416 mV) (Fig. 2b), and high mucoadhesive strength (35.64 ± 0.548%) (Table 2).

### Evaluation of the optimized FST-loaded LPHNPs formula (F3)

#### Transmission electron microscope (TEM)

**Figure 3:**
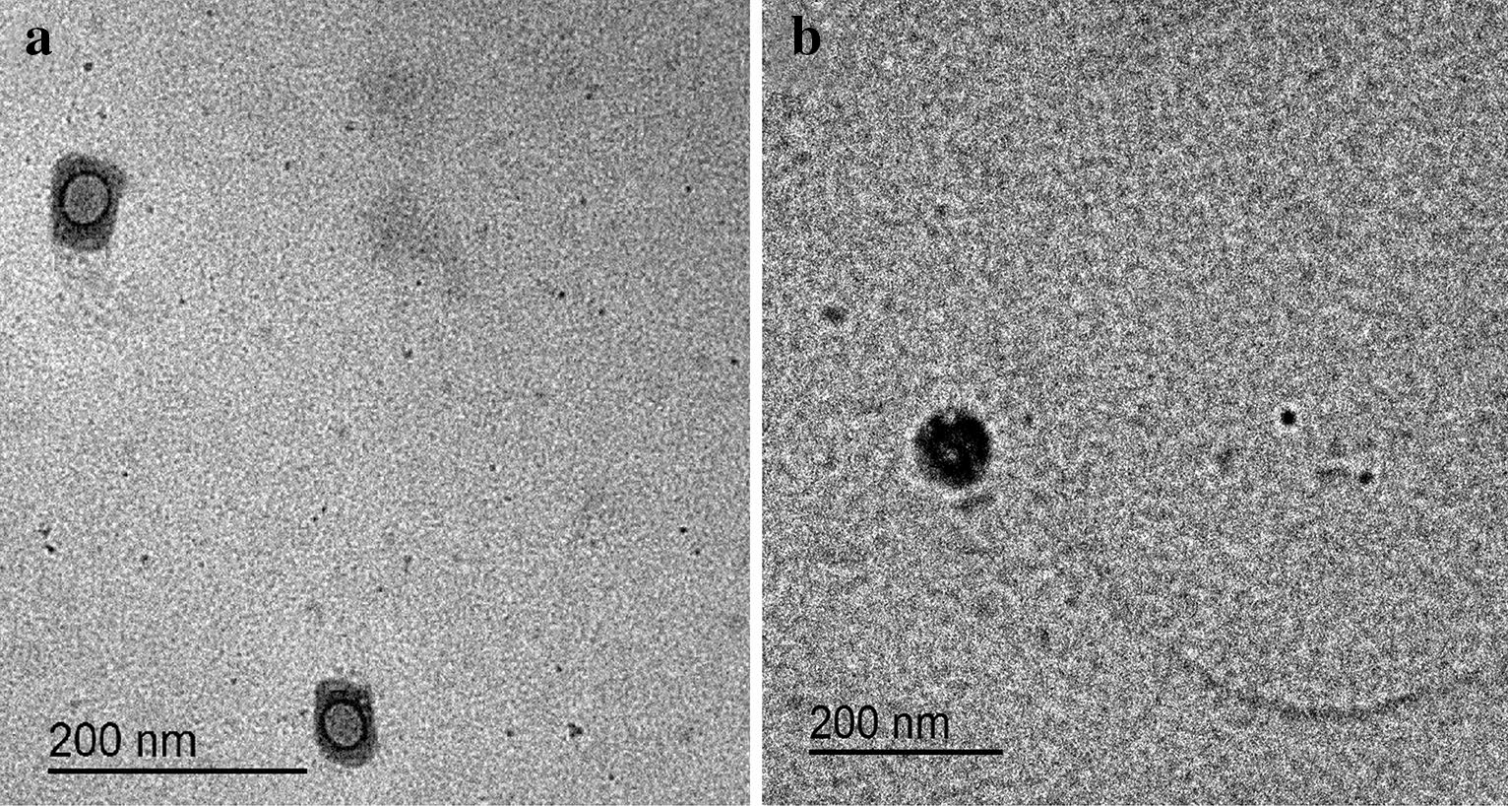
TEM images of (**a**) FST-loaded LPHNPs (F3) and (**b**) FST-uncoated NPs (F1).

TEM photograph of CT-coated LPHNPs formula (F3, Fig. 3a) displayed its spherical shape with a homogeneous monolayer CT coated at the periphery surrounding the lipid core encapsulating the drug in comparison with the uncoated one (F1, Fig. 3b)^37^. The size of particles measured by TEM were in accordance with those measured by DLS.

#### Fourier transform infrared spectroscopy (FT-IR)

**Figure 4:**
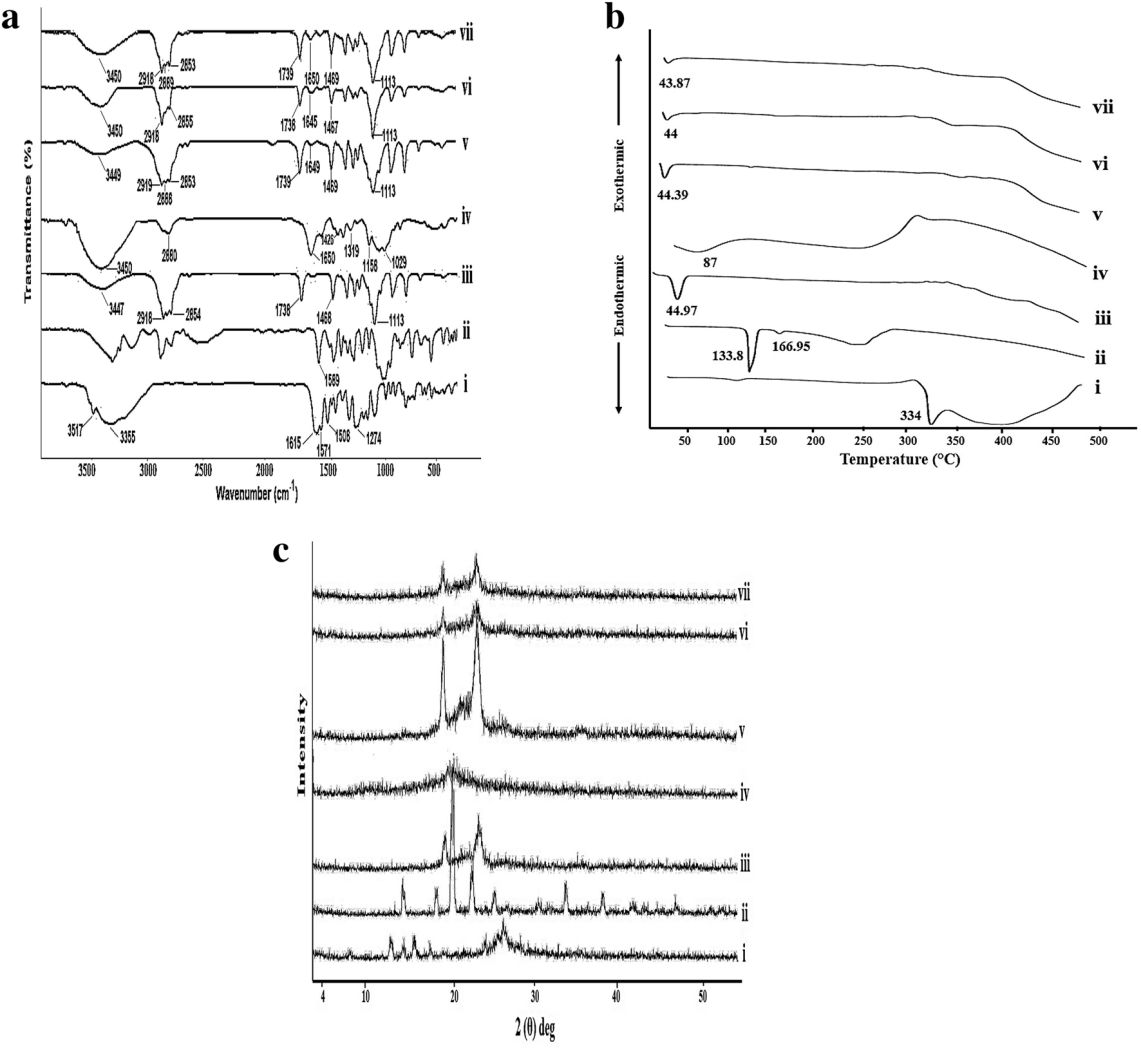
Solid characterization. (**a**) FT-IR spectra, (**b**) DSC thermograms, and (**c**) XRD patterns of pure FST (i), Tris (ii), Gelucire (iii), CT (iv), physical mixture corresponding to the optimal FST-loaded LPHNPs (F3) (v), Plain NPs (vi) and the optimal FST-loaded LPHNPs (F3) (vii).

The FT-IR spectra of LPHNPs (F3), as well as their ingredients are presented in Fig. 4a. The FT-IR spectrum of FST (i) demonstrated clear absorption bands at 3517 and 3355 cm^−1^ signifying the OH group^38^. The distinct absorption bands at 1615, 1571 and 1508 cm^−1^ were attributed to C=O, C–C, and C–O stretching vibration, respectively. The absorption band at 1274 cm^−1^ was attributed to C–O–H bending vibration^10^. A band at 1589 cm^−1^ regarding the bending vibration of the amino group appeared in the FT-IR spectrum of Tris (ii)^23^. The FT-IR spectrum of Gelucire (iii) showed two characteristic bands at 2854 and 1113 cm^−1^ related to C-H stretching vibration and C–O (ether) stretching vibration, respectively^39^. Also, two distinct bands appeared at 2918 and 1738 cm^−1^ owing to hydroxyl and carbonyl groups stretching, respectively. Moreover, the band corresponding to the C–H deformation of the alkyl group appeared at 1468 cm^−1^^40^.

CT spectrum (iv) elicited an overlapped broad peak at 3450 cm^−1^ appointed to stretching vibrations of amino and hydroxyl groups. Besides, a weak band of –CH stretching appeared at 2880 cm^−1^^17^. The absorption band at 1650 cm^−1^ denoted carbonyl stretching vibration of the secondary amide group^23^. Additionally, –CH_2_ bending and C–O stretching are established by the bands at 1426 and 1029 cm^−1^, respectively^2^. The peak at 1158 cm^−1^ was assigned to the asymmetric stretch of C–O–C. Also, the peak for C-N stretching vibration of type I amine was observed at 1319 cm^−1^^41^.

The spectra of the physical mixtures representing F3 (v) showed the bands of Gelucire, whereas those of FST and Tris disappeared. Besides, the intensities of CT bands were reduced in the case of the physical mixture spectrum corresponding to F3 (v). The aforementioned results may be ascribed to the dilution effect.

The spectra of the plain and medicated LPHNPs representing F3 (vi and vii, respectively) showed a similar pattern with a reduction in the intensity of bands in the region of 3000–3500 and 1000–1500 cm^−1^, which might be resulted from the overlapping of CT and Gelucire^42^. The spectra of the medicated formula [F3 (vii)] showed the absence of the FST bands verifying the entrapment of FST within NPs. Also, this assures no interaction between FST and other ingredients.

#### Differential scanning calorimetry (DSC)

The DSC thermograms of FST, Tris, Gelucire, CT, each alone and their physical mixture, and the medicated LPHNPs (F3) are shown in Fig. 4b.

Regarding FST, a distinctive endothermic peak was observed at 334 °C representing its melting point (i)^10^. For pure Tris (ii), a sharp endothermic peak at 166.95 °C related to the reported melting point values (168–172 °C). Another peak corresponding to its solid–solid transition appeared at 133.8 °C^23^. Gelucire (iii) exhibited a characteristic endothermic peak at 44.97 °C^43^. The broad CT peak at 87 °C (iv) was accredited to the polymer dehydration and water loss related to the hydrophilic nature of the amorphous CT due to the presence of the –OH group^23^.

Only one endothermic peak of Gelucire with prominent disappearance of those of FST, Tris and CT was spotted in the DSC thermogram of the physical mixture representing F3 (v). This may be due to the dilution effect.

Intriguingly, thermograms of both plain and medicated LPHNPs representing F3 (vi and vii, respectively) were similar to each other. They showed absence of the Tris characteristic peaks. Moreover, the vanishing of the FST peak was obvious in the thermogram of the medicated LPHNPs (vii), proposing FST entrapment in the LPHNPs matrix. Additionally, the disappearance of the CT peak might be due the peak masking by the relatively large Gelucire amount^23^.

#### X-ray diffractometry (XRD)

Figure 4c demonstrates that FST diffractogram (i) displayed strong diffraction peaks at 2θ of 12.65, 14.23, 15.53, 17.47, 24.19, and 28.5°, pointing out the crystalline nature of the drug^12^. In the case of Tris (ii), intense signals compliant with its intricate crystal lattice arrangement were detected at 2θ of 14°, 18.29°, 20.26°, 22.62° and 25.38°^44^. Gelucire 50/13 also displayed some crystallinity as shown by the two characteristic peaks of high intensity at 19.3° and 23.33 at 2θ (iii)^45^. Chitosan (iv) exhibited no distinctive diffraction peaks representing its amorphous nature^46^.

The physical mixtures representing F3 (v) showed the peaks of Gelucire with an absence of FST, Tris, and CT. The diffractogram of the medicated LPHNPs (F3) (vii) concurred with that of the plain one (vi) and showed absence of the characteristic FST peaks. This points out the drug encapsulation within the matrix of the NPs in either a molecular dispersed or amorphous state. Moreover, this indicates that the addition of FST did not change the nature of the NPs.

### In vitro release study

**Figure 5:**
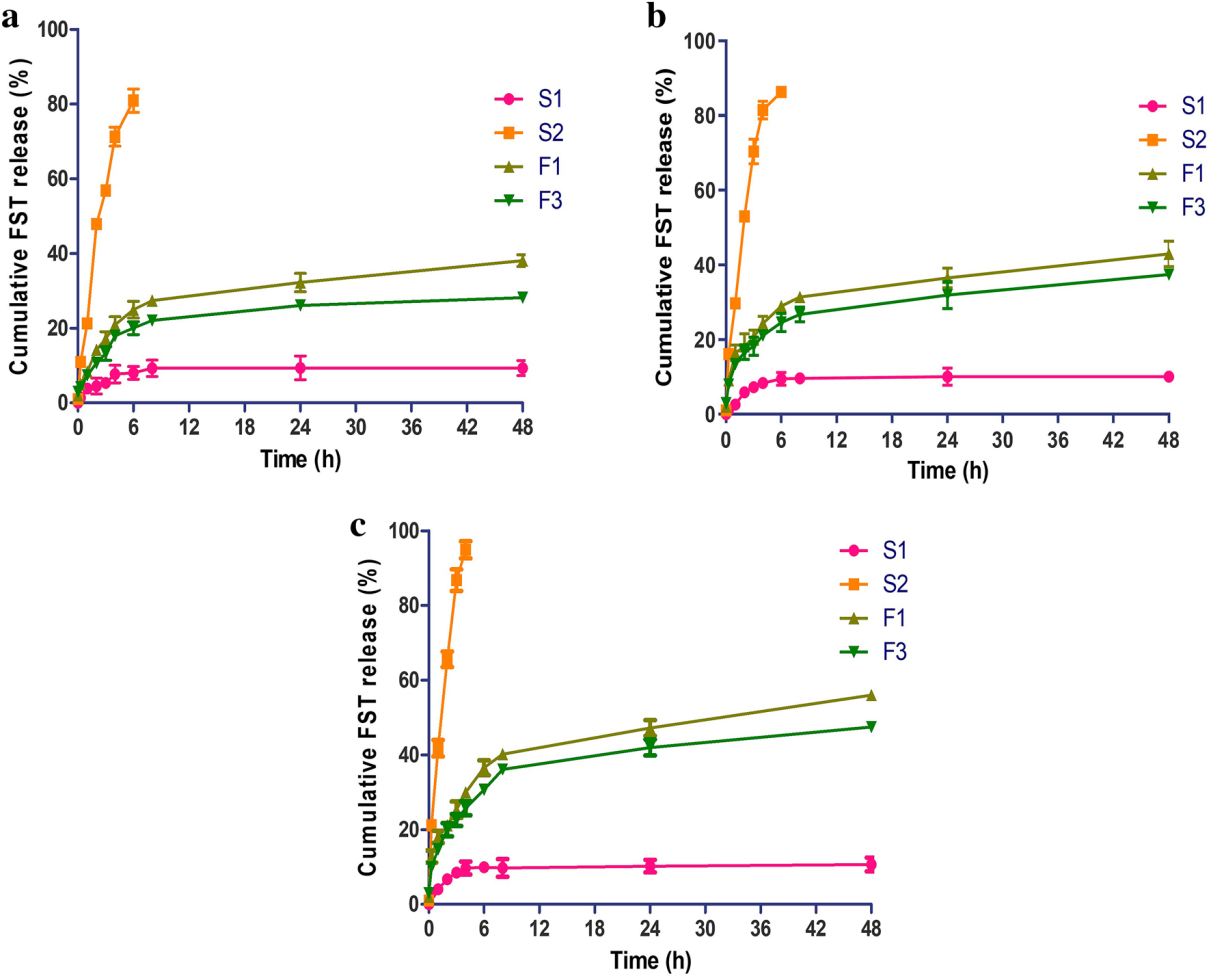
The in vitro release pattern of FST from FST-loaded Uncoated NPs (F1) and the optimal FST-loaded LPHNPs (F3) in comparison with its diffusion from aqueous FST dispersion (S1) and FST/Tris aqueous solution (S2) at three different pH media (**a**) pH 1.2, (**b**) pH 6.8, and (**c**) pH 7.4. Each point represents the mean ± SD (n = 3).

The in vitro release pattern of FST from FST aqueous dispersion (S1), FST/Tris solution (containing 6%w/v Tris) (S2), FST-loaded LPHNPs, and FST-loaded uncoated NPs (F3 & F1, respectively) in different media were compared (Fig. 5). Interestingly, a significant (*p* < 0.05) increase in the release rate was noticed for FST from FST/Tris (S2), FST-loaded uncoated NPs (F1), and FST-loaded LPHNPs (F3) in HCl of (pH 1.2), phosphate buffer of (pH 6.8 and 7.4) as compared to aqueous FST dispersion (S1). The rate of dissolution of FST from S2 was increased by about 7.81, 8.49, and 10.58 folds in HCl of (pH 1.2), phosphate buffer of (pH 6.8), and phosphate buffer of (pH 7.4) respectively, after 48 h as compared to its aqueous dispersion form (S1). This result highlights the successful improvement in the dissolution rate of FST with Tris.

Figure 5 shows that the highest release rate occurred in a phosphate buffer of (pH 7.4)^47^. This might be explicated by the fact that the hydroxyl groups of the FST molecule becomes electronegative in the alkaline medium inducing its water solubility, hence its dissolution rate^48^.

Circumspect inspection of Fig. 5 revealed that FST released from F3 and F1 in a sustained manner over 48 h. The effect of CT coating on the in vitro release behavior cannot be ignored. CT has a bimodular effect on the release pattern based on the pH of the release medium. In acidic conditions (Fig. 5a), the CT coat (F3) caused decrease in the release rate compared to the uncoated formula (F1). This might be attributed to the formation of a viscous layer in the microenvironment around the nanoparticles. This assures no dose dumping from the prepared LPHNPs. In alkaline conditions (pH 6.8 and 7.4) (Fig. 5b,c), the amino group of CT presents in the non-ionized form, preventing the formation of the viscous layer. Alternatively, CT coating will become swollen and foster the release of the entrapped FST^49^. Ultimately, the prepared FST-loaded LPHNPs (F3) might be regarded as nanoscaffolds for sustaining oral drug delivery systems.

### Kinetic analysis

The kinetic release data of FST -either free (dispersion and solution in Tris, S1 and S2, respectively) or encapsulated in NPs (coated and uncoated one, F3 & F1, respectively) in different release media were best fitted to Higuchi-diffusion model. Thus, diffusion-controlled drug signifies the rate-determining step as shown in Table 3. Furthermore, complementary analysis by Korsmeyer-Peppas and Weibull models confirmed a Fickian mechanism, since n is < 0.5 and β is ≤ 0.75, respectively, illuminating that the drug release was extensively controlled by diffusion. It was reported that other drugs loaded in LPHNPs followed similar kinetic patterns^50^.

**Table 3:** Mathematical modeling and release kinetics of the prepared FST-loaded LPHNPs (F3) and uncoated one (F1) as well as aqueous FST dispersion (S1) and FST/Tris aqueous solution (S2) (see Table 1 for F1 & F3 composition). (R^2^): Coefficient of determination, (n): Diffusional exponent, (β): shape parameter.

Formula code	Zero-order (R^2^)	First-order (R^2^)	Higuchi-diffusion model (R^2^)	Korsmeyer-Peppas equation		Mechanism of release	Weibull	
				(R^2^)	(n)		(R^2^)	(β)
F3 in (pH 1.2)	0.6292	0.6645	0.8408	0.8635	0.5076	Fickian	0.8743	0.6293
F3 in (pH 6.8)	0.5851	0.6261	0.8046	0.8808	0.3626	Fickian	0.8878	0.3971
F3 in (pH 7.4)	0.6169	0.6815	0.8485	0.9265	0.3753	Fickian	0.9367	0.4258
F1 in (pH 1.2)	0.6274	0.6787	0.8461	0.8926	0.4906	Fickian	0.9048	0.5324
F1 in (pH 6.8)	0.5978	0.6618	0.8177	0.8666	0.3385	Fickian	0.8783	0.3817
F1 in (pH 7.4)	0.5803	0.6598	0.8204	0.9023	0.3193	Fickian	0.9145	0.3752
S1 in (pH 1.2)	0.2371	0.2360	0.4065	0.5210	0.3540	Fickian	0.5208	0.3632
S1 in (pH 6.8)	0.2751	0.2766	0.4949	0.6858	0.4245	Fickian	0.6865	0.4350
S1 in (pH 7.4)	0.2333	0.2347	0.4358	0.5937	0.2532	Fickian	0.5919	0.2620
S2 in (pH 1.2)	0.3060	0.3571	0.5681	0.9724	0.4148	Fickian	0.7807	0.5704
S2 in (pH 6.8)	0.2693	0.3302	0.5290	0.9844	0.3407	Fickian	0.7666	0.5111
S2 in (pH 7.4)	0.2369	0.3424	0.4908	0.9866	0.2944	Fickian	0.7587	0.5542

### Stability study of the optimized LPHNPs (F3)

Table 4 illustrates the storage evaluation parameters data of the optimized FST-loaded LPHNPs formula (F3) at ambient and refrigerated (4 ± 1 °C) conditions. The optimized FST-loaded LPHNPs (F3) exhibited good stability which is verified by the absence of physical changes in either odor or color during the storage period (6 months) at (4 ± 1 °C). However, at the end of ambient storage condition, turbidity was noticed in the formulation.

**Table 4:** PS, PDI, ZP and drug retention % of FST-loaded LPHNPs aqueous dispersions (F3) stored at refrigerated (4 ± 1 °C) and ambient conditions (See Table 1 for F3 composition). Each value represents the ± SD (n = 3). * Significant at p < 0.05 monthly vs. initial, ** Highly significant at p < 0.01 monthly vs. initial, *** Extremely significant at p < 0.001 monthly vs. initial, ### Extremely significant at p < 0.001 refrigerated temperature vs. ambient temperature after 6 months.

Storage time	Evaluation parameters							
	Refrigerated temperature (4 ± 1 °C)				Ambient temperature			
	PS (nm)	PDI	ZP (mV)	Drug retention (%)	PS (nm)	PDI	ZP (mV)	Drug retention (%)
Zero time	125.39 ± 0.924	0.357 ± 0.012	+ 30.16 ± 1.418	100.00 ± 0.000	125.39 ± 0.924	0.357 ± 0.012	+ 30.16 ± 1.41	100.00 ± 0.000
1 month	119.42 ± 0.716	0.351 ± 0.014	+ 29.82 ± 1.722	99.72 ± 4.573	137.19 ± 2.709	0.382 ± 0.078	+ 25.56 ± 3.006	92.07 ± 1.226
2 month	118.67 ± 2.082	0.334 ± 0.028	+ 30.09 ± 2.610	94.34 ± 0.566	147.05 ± 0.172***	0.359 ± 0.055	+ 22.51 ± 2.789**	86.33 ± 8.572
3 month	121.33 ± 2.041	0.311 ± 0.005	+ 28.67 ± 3.396	93.05 ± 2.218	184.16 ± 4.092***	0.588 ± 0.132**	+ 20.99 ± 0.985***	84.44 ± 2.673
4 month	117.33 ± 3.451	0.412 ± 0.004	+ 28.54 ± 1.592	92.86 ± 4.962	205.75 ± 4.549***	0.608 ± 0.084***	+ 20.27 ± 0.231***	74.63 ± 6.302*
5 month	123.05 ± 3.595	0.342 ± 0.0.03	+ 30.29 ± 1.041	92.11 ± 0.247	223.00 ± 2.564***	0.673 ± 0.071***	+ 18.35 ± 2.394***	72.59 ± 5.711**
6 month	120.95 ± 1.624^###^	0.366 ± 0.038^###^	+ 29.96 ± 0.613^###^	90.42 ± 4.892^###^	239.45 ± 3.743***	0.733 ± 0.044***	+ 14.04 ± 1.977***	67.81 ± 4.267**

The statistical analysis of the drug retention %, PS, PDI, and ZP disclosed an insignificant difference during storage at refrigerated conditions (4 ± 1 °C). Contrary, in the case of ambient storage conditions both PS and PDI exhibited a significant (*p* < 0.05) increase, whereas ZP represented a significant (*p* < 0.05) decrease throughout the whole study. Regarding drug retention (%), it was in the acceptable range during the first 3 months of storage with subsequent significant (*p* < 0.05) downfall from the 4th month till the end of the stability study at ambient temperature^51,52^. The aforementioned data highlights the superior stability of the optimized FST-loaded LPHNPs (F3) at refrigerated temperature emphasizing its effectiveness over a prolonged time of storage for six months. Similar upshots concerning the storage stability examination of LPHNPs at the same temperatures were previously reported^53^.

### In vivo assessment studies of the optimized LPHNPs (F3)

#### Effect of the optimal FST-loaded LPHNPs (F3) on the pancreatic tissue

The condition of SAP is associated with a faulty release of large quantities of digestive enzymes from the acinar cells and their premature activation. As a result, the pancreatic tissue is subjected to severe injury followed by a late-stage of inflammatory response. In our study, a reported dose of l-arg was used to experimentally provoke this response in rats and cause SAP (250 mg/100 g)^25^. Selectively, it damages the pancreatic acinar cells and directly causes necrotizing pancreatitis^54^.

**Figure 6:**
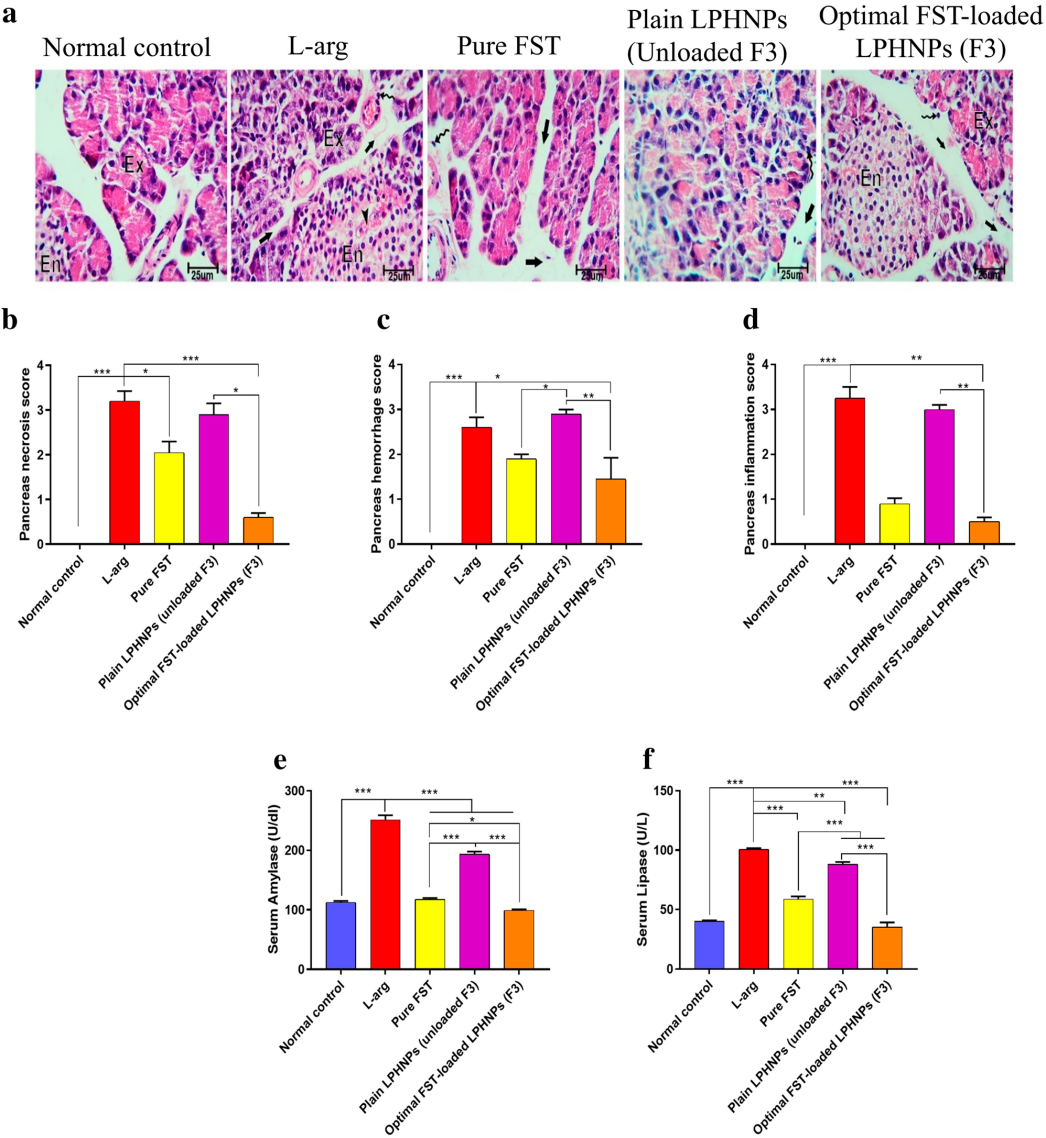
Effect of different experimental groups on l-arginine (l-arg) induced severe acute pancreatitis (SAP) in rats; (**a**) Histopathological changes of the pancreatic tissue stained with H&E. Black arrows, arrow heads and curved arrow reveal interstitial tissue edema, congested blood vessels and periductal inflammatory cell infiltration, respectively. Letters Ex and En show the exocrine and endocrine parts (Islets of Langerhans) of the pancreas, respectively. (**b**) Pancreas necrosis score, (**c**) Pancreas hemorrhage score, (**d**) Pancreas inflammation score, (**e**) Serum Amylase, (**f**) Serum Lipase. Data are represented as median and interquartile change in (**b**–**d**) and mean ± SEM in (**e**,**f**) (n = 6). *,**,*** significantly difference at *p*-value < 0.05, 0.01 and 0.001, respectively, using Kruskal–Wallis test followed by Dunn multiple comparison test (**b**–**d**) and One-way ANOVA followed by Tukey’s multiple comparisons test (**e**,**f**). (Magnification X: 400, bar = 25 μm).

Pancreatic tissue of the normal group showed normal architecture conferring both the endocrine and exocrine parts (Islets of Langerhans) of the pancreas (Fig. 6a). The acini showed basal basophilia and apical acidophilia separated by minimal connective tissue stroma (Fig. 6a). On the other hand, the l-arg group revealed a clear disruption of pancreatic architecture with the individual acini, having week basal basophilia and apical acidophilia, interstitial tissue edema, congested blood vessels, acinar cell necrosis and periductal inflammatory cell infiltration (Fig. 6a). The endocrine part showed disrupted pale cells and injury, a semi-quantitation of pancreatic necrosis, hemorrhage, and inflammation indicating a significant increase in their scores in the l-arg group when compared with the normal group (Fig. 6b-d). In addition, to verify a successful induction of the model, serum amylase and lipase levels, two golden diagnostic markers of SAP, were measured disclosing a significant elevation of both in the l-arg group by 2.2- and 2.5-folds, respectively, when compared to the normal group (Fig. 6e,f).

It is reported that FST demonstrated the ability to protect against cerulein-induced acute pancreatitis (AP)^24^. In agreement, administration of FST either alone or loaded into LPHNPs (F3) exhibited focally decreased edema, acinar vacuolization, inflammatory cell periductal neutrophil infiltration, as well as acinar cell necrosis. The Islets of Langerhans appeared less affected, as well. Interestingly, optimal FST-loaded LPHNPs formula (F3) was superior in triggering the histopathological alterations (Fig. 6a), as evidenced by the histopathological scorings (Fig. 6b–d), as well as by the activities of both amylase and lipase enzymes (Fig. 6e,f) compared to the groups administered l-arg alone, pure FST or plain LPHNPs (unloaded F3).

#### Anti-oxidant effect of the optimal FST-loaded LPHNPs (F3) on l-arg-induced pancreatic oxidative stress

**Figure 7:**
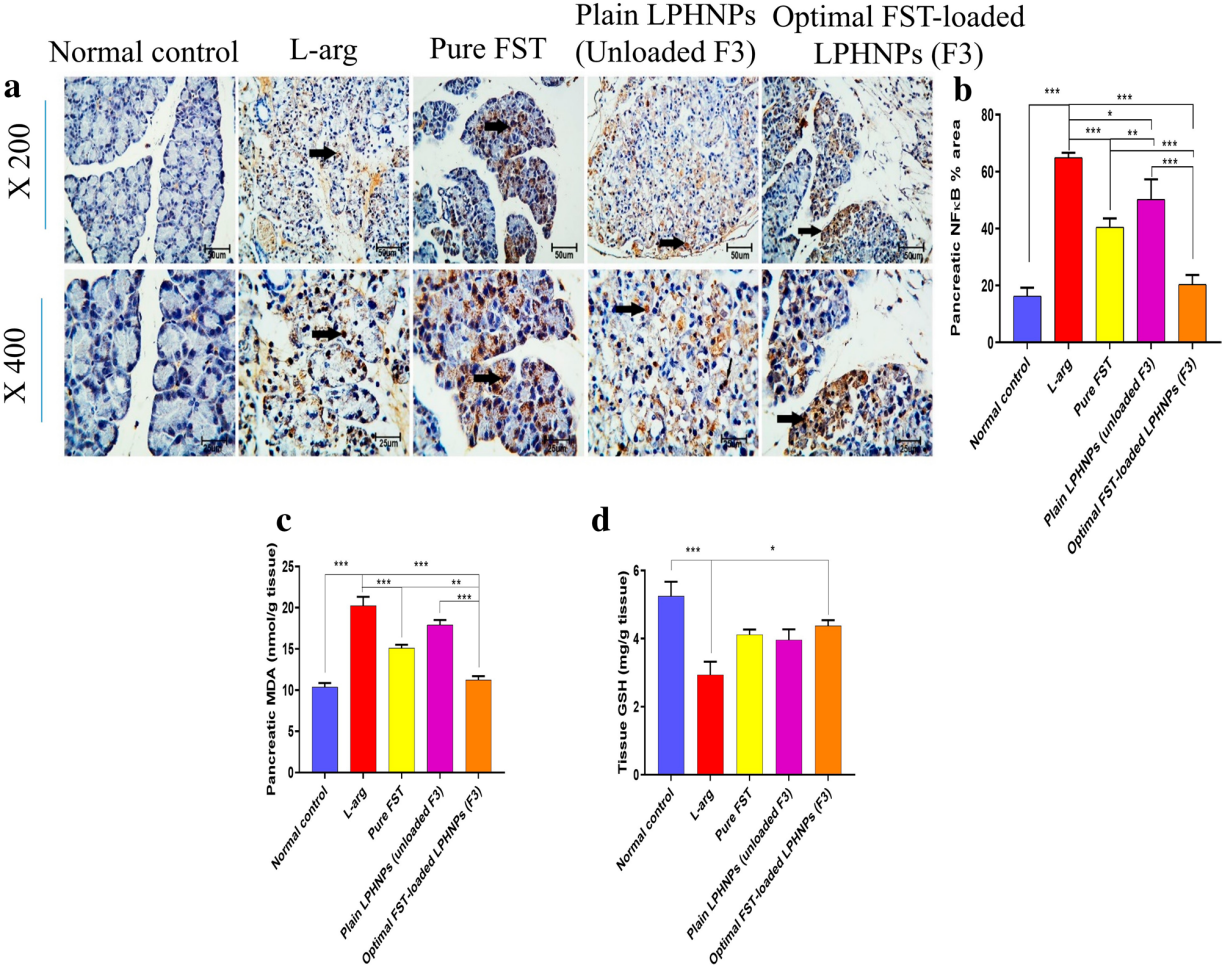
Anti-oxidant effect of the optimal FST-loaded LPHNPs on l-arg-induced pancreatic oxidative stress in rats; (**a**) Immunohistochemical (IHC) staining of nuclear factor-κB (NF-κB). Black arrow indicates nuclear staining. (**b**) Pancreatic NF-κB % area, (**c**) Malondialdehyde (MDA) level, (**d**). Reduced glutathione (GSH) level, Data are represented as mean ± SEM (n = 6). *,**,*** significantly difference at *p*-value < 0.05, 0.01 and 0.001, respectively, using One-way ANOVA followed by Tukey’s multiple comparisons test (**b**–**d**). (Magnification X: 200, bar = 50 μm, X: 400, bar = 25 μm).

The activation of NF-κB has been regarded as a key event in the progression of NF-κB which is triggered via the overly generated reactive oxygen species (ROS)^55^. Just after activation of NF-κB, it travels from the cytoplasm to the nucleus to behave as a transcription factor for several proinflammatory cytokines. Interestingly, the level of NF-κB has been related to the severity of AP leading to chronic pancreatitis eventually. In doing so, we measured the level of NF-κB by IHC (Fig. 7a,b). The l-arg group demonstrated a significant elevation of NF-κB in comparison with the normal group, showing nuclear immunostaining, indicating increased activity.

It has been reported that FST acted as an antioxidant thus causing the reduction of NF-κB activity^56^. In our study, pretreatment with FST, either alone or loaded in LPHNPs (F3), significantly reduced the NF-κB level compared to the l-arg group and significantly reduced its nuclear translocation.

The pathogenesis and progression of AP are closely related to an augmentation of the oxidative stress response of the pancreas^57^. Indeed, oxidative stress acts as a link between apoptosis and inflammation. First, we investigated the oxidative status by measuring the pancreatic MDA level, a known peroxidation product. The level of MDA was significantly elevated in the l-arg group compared to the Normal control group (Fig. 7c). Furthermore, the level of GSH was significantly reduced in the l-arg group in comparison with the Normal control group (Fig. 7d). Treatment with FST, either alone or nanoparticles (F3), reduced the level of MDA in the pancreatic tissue compared to the l-arg group (Fig. 7c). Notably, pretreatment with FST-loaded LPHNPs (F3) not only managed to reduce the level of MDA but also the nanoparticles spared the consumption of GSH causing a significant elevation of its level as compared to the l-arg group (Fig. 7d).

#### Impact of the optimal FST-loaded LPHNPs (F3) on l-arg-induced pancreatic inflammation

Inflammation has been a significant aspect of the pathogenesis of AP. Adipose lipolysis occurs as a result of pancreatic lipase interstitial leak, hence, releasing toxic amounts of unsaturated fatty acids. They, in turn, trigger an excessive release of inflammatory markers, which may lead to the disease progressing and eventually leading to multi-organ failure^58^. The involvement of the innate immune system is profound in motivating pathogenesis and contributes to disease development^59^. TLR4 is a receptor of the innate immunity involved with the recognition of pathogen- or damage-related molecular patterns. TLR4-mediated signaling pathway triggers the activation of NF-κB which induces the transcription and the release of many inflammatory mediators and cytokines^60^.

**Figure 8:**
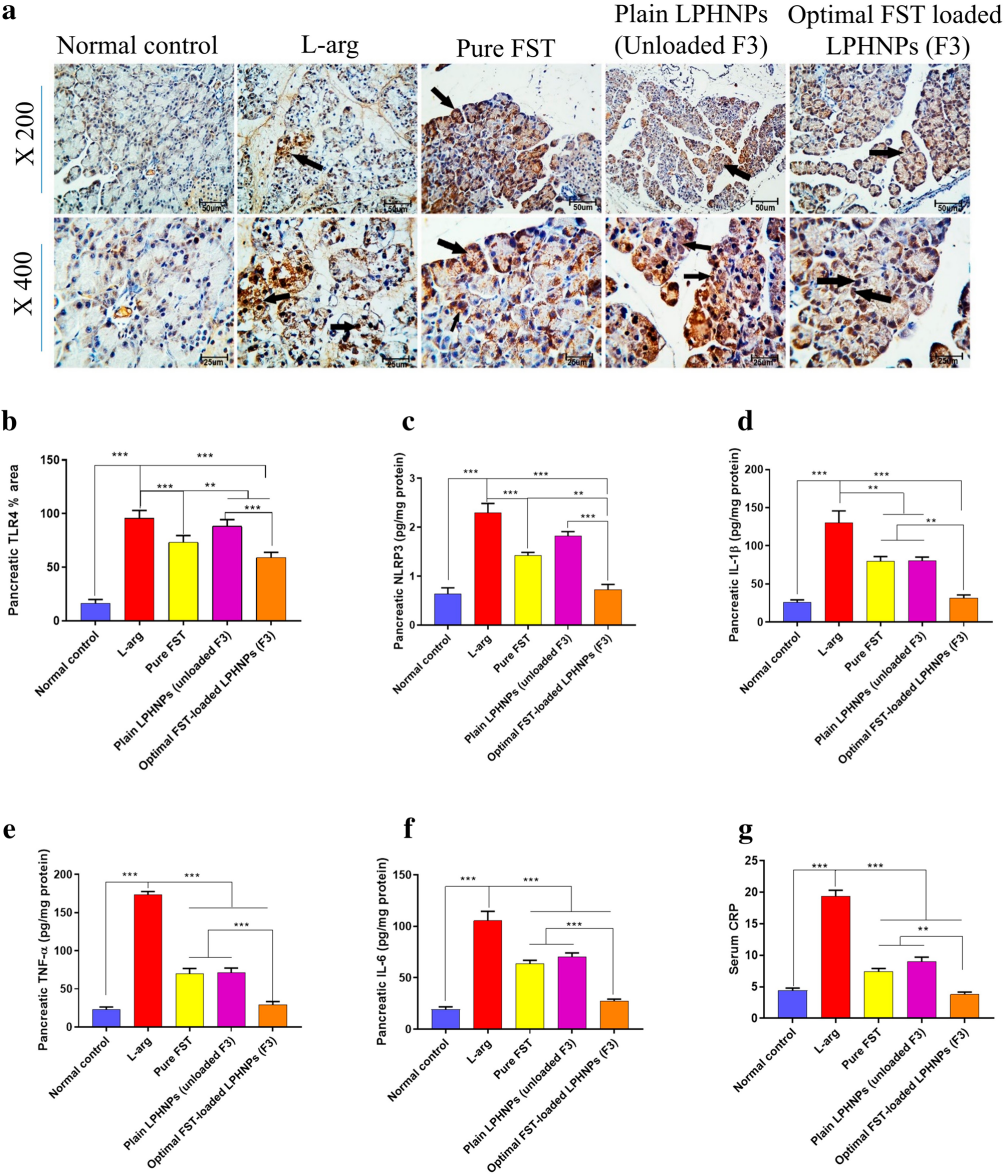
Anti-inflammatory effect of different experimental groups on l-arg-induced pancreatic inflammation in rats; (**a**) Immunohistochemical (IHC) staining of Toll-like receptor 4 (TLR4). Black arrow indicates Positive staining. (**b**) Pancreatic TLR4% area, (**c**) Pancreatic nucleotide-binding oligomerization domain, leucine-rich repeat and pyrin domain-containing protein 3 (NLRP3) level, (**d**) IL-1β, (**e**) TNF-α, (**f**) IL-6, (**g**) Serum C-reactive protein (CRP), Data are represented as mean ± SEM (n = 6). **,*** significantly difference at *p*-value < 0.01 and 0.001, respectively, using One-way ANOVA followed by Tukey’s multiple comparisons test (**b**–**g**). (Magnification X: 200, bar = 50 μm, X: 400, bar = 25 μm).

Figure 8a,b showed a significant elevation in TLR4 protein level in the l-arg group in comparison with the normal group. Contrarily, treatment with FST-loaded LPHNPs (F3) significantly reduced the level of TLR4 compared to either l-arg or Plain LPHNPs-treated groups.

It is reported that NF-κB affects the levels and expression of many proinflammatory cytokines. Hence, we measured NLRP3, an intracellular receptor closely related to pyroptosis, IL-1β, TNF-α, and IL-6 (Fig. 8c–f). All the aforementioned inflammatory mediators were elevated significantly in the l-arg group compared to normal. Pretreatment with FST either alone or loaded in LPHNPs (F3), revealed a significant decrease in pancreatic tissue inflammation compared to the l-arg group. Conspicuously, FST-loaded LPHNPs (F3) exhibited the most significant reduction of inflammation compared to the other investigated groups.

Abdelmageed et al.^27^, measured the level of C-reactive protein (CRP) as an indicator of l-arg induced AP. Figure 8g illustrates a significant elevation in the CRP level in the l-arg group indicating a severe inflammation compared to the normal group. Fruitfully, FST-loaded LPHNPs (F3) demonstrated the most significant decrease in the level of CRP compared to the other groups, except for the normal group, indicating the successful anti-inflammatory efficacy of FST when formulated as loaded LPHNPs.

Such conspicuous pancreatic protective efficacy may be accredited to several factors. First of all, FST offers a pharmacological influence on pancreatic abnormalities due to its anti-inflammatory and anti-oxidant properties^24^. Also, CT mucoadhesive properties may prolong the LPHNPs residence time, sustaining the release of FST and decreasing dosage frequency^61^. Fortunately, the permeability-boosting characteristics of CT could enhance the transcellular and paracellular uptake of LPHNPs by reversibly unlocking tight junctions across and between epithelial cells^62^. Conclusively, encapsulation of FST into LPHNPs coated with positively charged CT may be potentially considered as an effective way to target several touchpoints with one agent which can dampen pancreatic tissue damage, inflammatory cascade, and immune cell recruitment.

#### Impact of the optimal FST-loaded LPHNPs (F3) on lung injury

**Figure 9:**
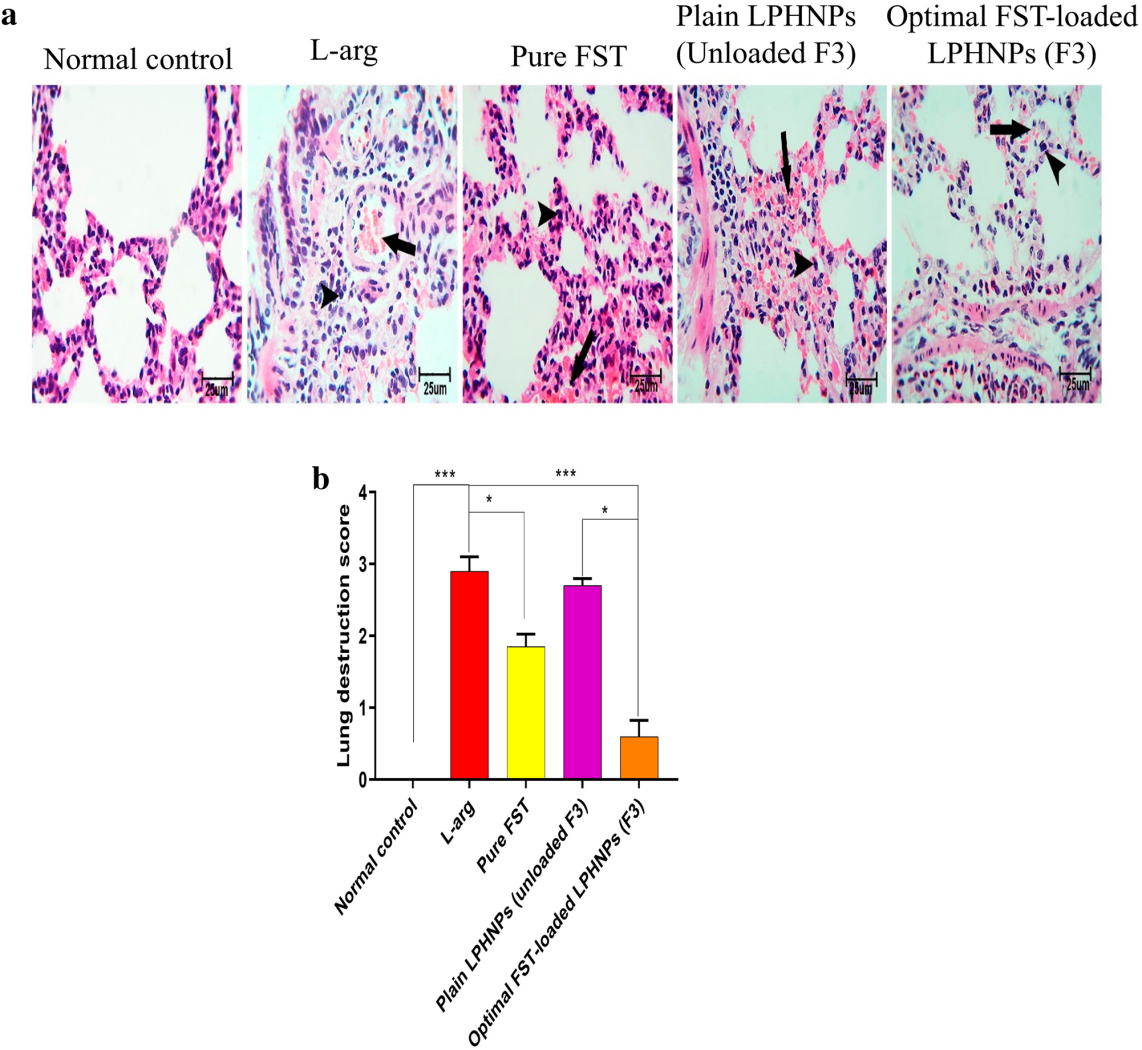
Impact of different experimental groups on l-arginine (l-arg)-induced pulmonary injury in rats; (**a**) Histopathological changes of the pulmonary tissue stained with H&E. Black arrows and arrow heads point to congestion and perivascular leukocytic cell infiltration, respectively. (**b**) Pulmonary destruction score. Data are represented as median and interquartile change, (n = 6). *, *** significantly difference at *p*-value < 0.05 and 0.001, respectively, using Kruskal–Wallis test followed by Dunn multiple comparison test (**b**), (magnification X: 400, bar = 25 μm).

As previously reported, most of the death cases among non-admitted SAP have been associated with severe lung injury^63^. Even upon hospital admission, almost 50% of patients die within the first week from multiple organ failures including respiratory failure^64^. Moreover, the survivors often suffer from a worsened quality of life^65^. To enrich more what has been perceived in our study on AP, we investigated the effect of the optimal FST-loaded LPHNPs (F3) on lung injury associated with l-arg-induced SAP. Normal lung tissue, stained by H&E, from the control rats exhibited a normal arrangement of alveoli and interalveolar septae with a clear cavity (Fig. 9a).

In the l-arg group, the protein-rich exudate eventually leaked into the alveolar space and interstitial tissues as a result of the marked blood vessel congestion in the lung tissue. Moreover, thickening of the interalveolar septa and perivascular, and interstitial leukocytic cell infiltration were observed in the l-arg group (Fig. 9a) which is a known cause of the observed injury and breakdown of the pulmonary parenchyma^66^. FST, pure and loaded into LPHNPs (F3), reduced the induced pulmonary damages by exhibiting reduced histological alterations seen in the l-arg and Plain LPHNPs groups (Fig. 9a). Notably, the pulmonary destruction scoring of the groups revealed the most significant reduction to be in the optimal FST-loaded LPHNPs (F3) group (Fig. 9b).

#### Impact of the optimal FST-loaded LPHNPs (F3) on liver and kidney injuries

**Figure 10:**
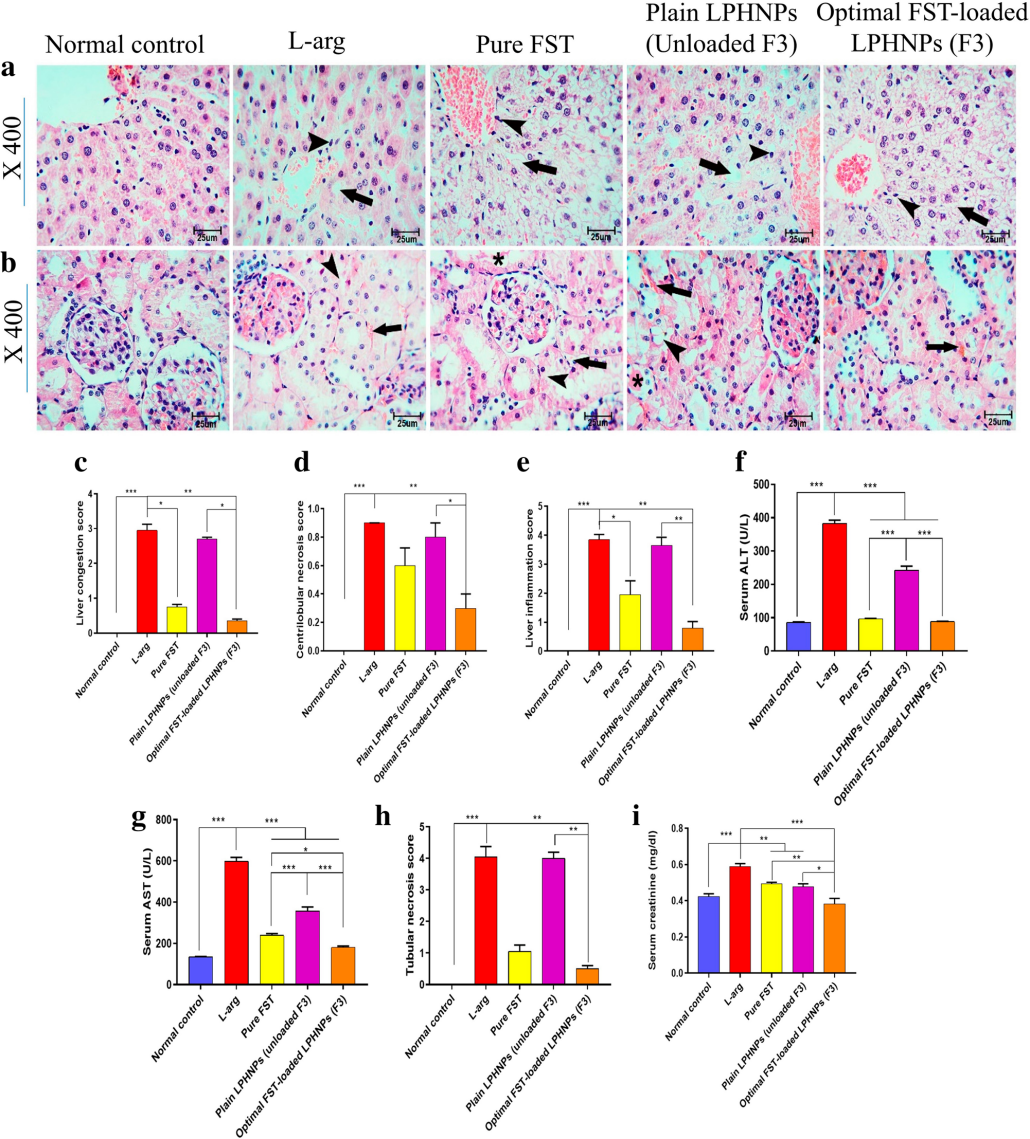
Impact of different experimental groups on l-arginine (l-arg)-induced hepatic and renal injury in rats; (**a**) Histopathological changes of the hepatic tissue stained with H&E, Black arrows and arrow heads point to vacuolar degeneration and periportal leucitic cell infestation. (**b**) Histopathological changes of the renal tissue stained with H&E, Black arrows, arrow heads and asterix point to congestion, tubular cell lining vacculation and intratubular cast deposition. (**c**) hepatic congestion score, (**d**) Hepatic necrosis score, (**e**) Hepatic inflammation score, (**f**) Serum alanine aminotransferase (ALT), (**g**) Serum aspartate aminotransferase (AST), (**h**) Tubular necrosis score, (**i**) Serum creatinine. Data are represented as median and interquartile change in (**c**–**e**) and H and mean ± SEM in (**f**,**g**,**i**) (n = 6). *,**,*** significantly difference at *p*-value < 0.05, 0.01 and 0.001, respectively, using Kruskal–Wallis test followed by Dunn multiple comparison test (**c**–**e**,**h**) and One-way ANOVA followed by Tukey’s multiple comparisons test (**f**,**g**,**i**). (Magnification X: 400, bar = 25 μm).

Persevering, we further examined both the hepatic and renal tissues of the studied groups. In (Fig. 10a,b, respectively) the Normal group exhibited a normal architecture of both liver and kidney. In contrast, the l-arg group revealed marked congestion of the central veins and sinusoids, along with vacuolar degeneration in the hepatocytes, as well as portal areas expansion with leukocytic cells periportal infiltration (Fig. 10a). Renal cortices sections from that group showed marked congestion of the interstitial tissue with tubular cell lining vacuolation (Fig. 10b). Both groups treated with FST (either pure or loaded in LPHNPs; F3) exhibited marked preservation of the two organs showing mild congested central ventral veins and vacuolar degeneration with limited leukocytic cells periportal infiltration and almost normal glomeruli with minimal tubular cell lining vacuolation (Fig. 10a,b). The hepato- and reno-protective effects of FST-loaded LPHNPs (F3) were evidenced by the hepatic congestion, necrosis, and inflammation scores (Fig. 10c–e) and the tubular necrosis score (Fig. 10h).

Finally, serum markers of both hepatic and renal injury were measured as ALT (Fig. 10f), AST (Fig. 10g) activities, and creatinine level (Fig. 10i). All three biomarkers were significantly high in the l-arg group. Both ALT and AST are intracellular enzymes that are released upon cellular damage; ALT reflects more acute hepatic necrosis whereas AST reflects possible injury in the liver and other organs^26^. FST, either alone or nanoformulation (F3), was able to spare the liver from such injury and thus significantly preventing the elevation of the enzymes in the serum compared to the l-arg and Plain LPHNPs.

In addition, the accumulation of creatinine, a non-protein nitrogenous byproduct, in the serum reflects a possible renal dysfunction. FST, either alone or nanoformulation (F3), was able to significantly reduce serum creatinine indicating a renoprotective effect. Interestingly, pretreatment with FST-loaded LPHNPs (F3) exhibited a significant reduction of both AST activity and creatinine level compared to those of the group administered FST alone. Previously, FST has been demonstrated to spare the pancreas in another model of induced AP^23^.

## Conclusions

Concisely, FST-loaded LPHNPs were successfully prepared through conjoined ultrasonication and w/o/w double emulsion techniques. The prepared NPs were evaluated regarding EE %, LC %, PS, PDI, ZP, and mucoadhesive strength to select the optimum one that was subjected to further characterization and extensive investigations. The results of FT-IR, DSC, and XRD for the optimized LPHNPs assured the FST entrapment within the LPHNPs matrix. TEM imaging of LPHNPs unveiled the nanosized structure with spherical morphology. The optimum LPHNP (F3) demonstrated physical stability at the refrigerated temperature. The obvious in vivo assessment of the optimal FST-loaded LPHNPs formula was declared by histopathological, biochemical studies, and IHC. These promising preclinical findings merit insightful attention for prospective clinical evaluation of the prepared phyto-pharmaceutical lipomer as a safe and efficient natural anti-inflammatory therapy for severe inflammation that may progress to cancer. Consequently, this novel phytopharmaceutical delivery system of FST may be considered an auspicious delivery one for sustaining the effectiveness of FST especially when a prolonged therapy is a must for SAP.

## Data Availability

All raw and analyzed data as well as the materials are available in this study.
